# Technical details of thoracic endovascular aortic repair with fenestrations for thoracic aortic pathologies involving the aortic arch: A Chinese expert consensus

**DOI:** 10.3389/fcvm.2022.1056229

**Published:** 2022-12-20

**Authors:** Chenyang Qiu, Zhenjiang Li, Xiangchen Dai, Xinwu Lu, Qingsheng Lu, Xiaoqiang Li, Weimin Zhou, Pingfan Guo, Jun Pan, Donglin Li, Ziheng Wu, Hongkun Zhang

**Affiliations:** ^1^Department of Vascular Surgery, The First Affiliated Hospital, School of Medicine, Zhejiang University, Hangzhou, China; ^2^Department of Vascular Surgery, General Hospital, Tianjin Medical University, Tianjin, China; ^3^Department of Vascular Surgery, Shanghai Ninth People’s Hospital, Shanghai Jiao Tong University School of Medicine, Shanghai, China; ^4^Department of Vascular Surgery, The First Affiliated Hospital of Naval Medical University, Shanghai, China; ^5^Department of Vascular Surgery, Nanjing Drum Tower Hospital, Affiliated to Nanjing University Medical School, Nanjing, Jiangsu, China; ^6^Department of Vascular Surgery, The Second Affiliated Hospital of Nanchang University, Nanchang, Jiangxi, China; ^7^Department of Vascular Surgery, The First Affiliated Hospital of Fujian Medical University, Fuzhou, China

**Keywords:** thoracic endovascular aortic repair, fenestrations, standard procedure protocol, technical details, device

## Abstract

Thoracic aortic pathologies involving the aortic arch are a great challenge for vascular surgeons. Maintaining the patency of supra-aortic branches while excluding the aortic lesion remains difficult. Thoracic EndoVascular Aortic Repair (TEVAR) with fenestrations provides a feasible and effective approach for this type of disease. The devices needed in the procedure are off-the-shelf, with promising results reported in many medical centers. Up until now, there have been no guidelines focusing exclusively on the details of the TEVAR technique with fenestrations. Experts from China have discussed the technical parts of both *in situ* fenestrations (needle and laser) and fenestrations *in vitro* (direction inversion strategy and guidewire-assisted strategy), providing a technical reference to standardize the procedure and improve its results.

## Introduction

In the past, open surgery has been the main approach for thoracic aortic pathologies involving the aortic arch. Thanks to the development of endovascular techniques and devices, an increasing number of patients suffering from this disease have begun to receive a different kind of treatment (1). The Chimney technique and branched stents are two strategies for pathologies involving the aortic arch. The Chimney technique presents a high risk of type I endoleaks, and the branched stent is limited by a lack of custom-made instruments. TEVAR with fenestrations has gradually become the most used approach in this field (2–4). Given that custom-made fenestrated stent grafts are not yet available in China, physician-made fenestrations represent the main treatment for thoracic aortic pathologies affecting the aortic arch. Aortic dissection and aneurysms can both be treated with TEVAR with physician-made fenestrations. According to reports from many medical centers, the equipment needed for such treatments is commercially available, and the results after applying this method are relatively promising. However, this technique’s limitations are due to the lack of a standard procedure protocol concerning the technical details of TEVAR with fenestrations (3, 5–8). As a result, experts from China have started to discuss the technical aspects of this operation and have agreed to establish a standard procedure reference.

## Standard TEVAR procedure with fenestrations

### 1 Vascular access

A stent graft is typically delivered through the femoral artery. The branches of the left brachial artery (LBA), left common carotid artery (LCCA), and right common carotid artery are frequently used for stent placement (9).

### 2 Angiography before stent graft deployment

A pigtail catheter is inserted via the left brachial artery into the ascending aorta, and an anterior-posterior aortic arch angiography is performed to assess the bilateral carotid and vertebral arteries. Then, a stiff guidewire (Lunderquist, Cook Medical, Bloomington, IN) is inserted via femoral artery access into the ascending aorta. A gold marker pigtail catheter is inserted through the stiff guidewire and positioned within the aortic arch. An angiography of the left anterior oblique aortic arch (between 45° and 65°) is performed to identify the arch architecture and location of the lesion; it is important to prevent the overlapping between the aortic arch and supra-aortic branch arteries in the left anterior oblique angiography (7).

### 3 *In situ* fenestrations

#### 3.1 Delivery and deployment of stent grafts

The aortic arch and branch openings are marked after an angiography of the aortic arch. Systolic pressure is reduced to between 90 and 100 mm Hg (7). The stent graft is delivered from the femoral access to the aortic arch through a Super Stiff guidewire (Lunderquist, Cook Medical). In order to avoid covering the LCCA, the proximal landing zone for fenestrations of the left subclavian artery (LSA) alone is the distal side of the opening of the LCCA. The proximal landing zone for LSA and LCCA fenestrations is located on the distal side of the opening of the brachiocephalic artery (BCA). The ascending aorta is the landing zone for fenestrations of all super-aortic stents. To prevent angulation between the stent graft and ascending aorta, the proximal portion of the stent should be as parallel as possible to the ascending aorta (8). Patients with supra-aortic branch fenestrations should receive additional cerebral blood supply through extracorporeal circulation (section “5 Assistive techniques and cerebral blood supply monitoring”).

#### 3.2 Fenestrations

##### 3.2.1 Needle fenestrations

**FIGURE 1:**
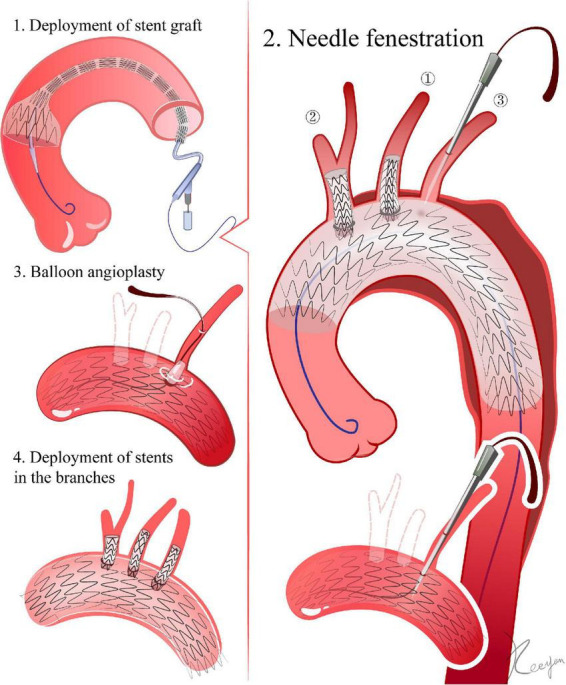
Needle fenestration. 1. Delivery of the stent graft. The ascending aorta is the landing zone for fenestrations of all super-aortic stents. 2. Needle fenestrations. In LSA, for example, a 6F angle-adjustable sheath is introduced retrogradely until its tip reaches the aortic stent graft. Once the sheath reaches the ideal place, a flexible needle is employed to create the fenestration in the aortic stent graft. A 0.018-inch guidewire is advanced through the needle’s aperture and into the ascending aorta after the puncture. 3. The initial aperture is then expanded by balloon angioplasty. 4. Deployment of stents in the branches.

LSA: From the LBA, a 6F angle-adjustable sheath (Lifetech, Inc., Shenzhen, China) is introduced retrogradely until its tip reaches the aortic stent graft. The tip is then adjusted to be as perpendicular as possible to the larger curve of the aortic stent graft. Once the sheath gets to the ideal position, a flexible needle (21 gauge, Futhrough, Lifetech, Inc.) is employed to create the fenestration in the aortic stent graft. Following the puncture, a 0.018-inch guidewire (V-18 ControlWire; Boston Scientific, Natick, MA) is inserted through the needle aperture and into the ascending aorta (1, 10, 11) (Figure 1).

LCCA and BCA: a short sheath is placed into the common carotid artery, with its tip reaching the membrane of the stent-graft. A needle (21G, 18 cm, BARD) is inserted through the sheath. Then, under fluoroscopy, a needle puncture is performed with a guidewire passing through the needle into the stent graft (6, 12–14).

##### 3.2.2 Laser-assisted fenestration

**FIGURE 2:**
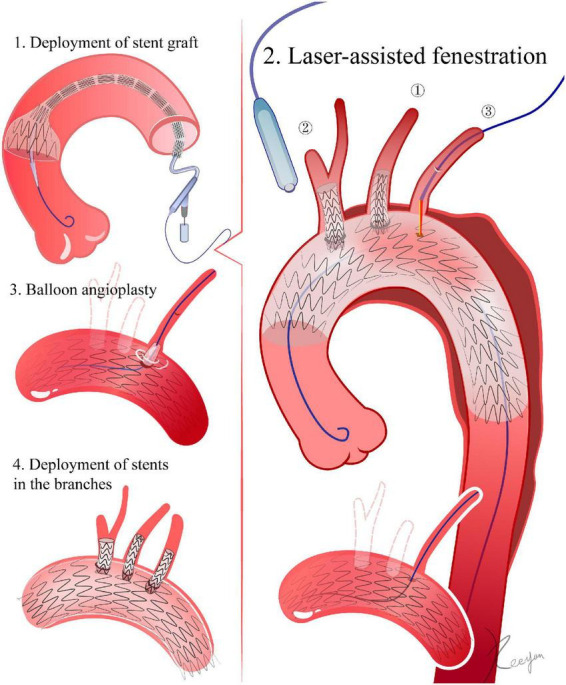
Laser-assisted fenestration. 1. Delivery of the stent graft. The ascending aorta is the landing zone for fenestrations of all super-aortic stents. 2. Laser-assisted fenestration. In LSA, for example, an angle-adjustable sheath is advanced until its tip touches the stent graft. The laser fiber then advances within the sheath until it gets to the membrane of the stent graft. Then, the laser machine is activated. After fenestration, a 0.018-inch guidewire is advanced through the initial aperture and into the ascending aorta. 3. The initial aperture is then expanded by balloon angioplasty. 4. Deployment of stents in the branches.

Laser preparation: the fiber energy can be tested *in vitro* in a moist environment its proximal end can be cut to 0.5 mm (810∼1,110 nm wavelength, pulsed type). A laser fiber and a balloon catheter are combined, with the fiber proximal end protruding 0.5–1 cm from the balloon catheter and connected by a Y connector (Merit Angioplasty Pack™, Merit Medical, Parkway, South Jordan, UT) (15, 16). Fenestration: For the LSA, an angle-adjustable sheath is advanced through the LBA. For the LCCA and BCA, a vascular sheath is inserted through the corresponding carotid artery. The sheath tip is pushed against the stent graft. The fiber and the balloon catheter are then inserted into the sheath until they reach the membrane of the stent graft. After activating the laser machine to deliver energy (18 W, pulsed, 2–3 times), the laser fiber and balloon catheter are pushed forward, and the fiber is withdrawn. If they are pushed inside the stent graft, the balloon catheter can be left inside (2, 17–20) (Figure 2).

#### 3.3 Balloon angioplasty

The initial aperture is then expanded by a 4 mm balloon, followed by the exchange of a 0.035-inch stiff guidewire (Amplatz, Boston Scientific) through the balloon catheter. The aperture is expanded by replacing the balloon catheter with one with a bigger diameter. The final size of the balloon depends on the diameter of the branch vessel. The size of the balloon is slightly smaller than the diameter of the corresponding artery, and a high-pressure balloon is more suitable during this process. If the aperture is resistant to balloon angioplasty, a cutting balloon (Boston Scientific) can be applied (21, 22).

#### 3.4 Stent deployment in the branches

LSA: it is preferable to use a self-expanding or balloon-expandable covered stent. If the LSA is distant from the lesion and the endoleak risk is low, a bare stent can be taken into consideration. The diameter of the stent should be the same (1–2 mm) or slightly greater than the diameter of the LSA opening. The stent should extend roughly 10 mm into the aorta. The distal end of the stent must not cover the vertebral artery’s orifice (1, 13, 14).

LCCA and BCA: similar to the balloon angioplasty procedure utilized for the LSA. The stent should extend roughly 10 mm into the aorta. The distal end of the stent should avoid the opening of the right common carotid artery during BCA stent deployment (12, 16, 22, 23).

#### 3.5 Sequence of fenestrations

For both LCCA and LSA fenestrations, the LCCA should be fenestrated first. When the stent deployment is complete in the LCCA, the sheath must be withdrawn and a pre-placed purse suture tightened to achieve hemostasis at the puncture site, therefore minimizing blood flow interference. The LSA fenestration should be performed last (10).

Regarding fenestrations for all supra-aortic branches, we recommend performing them in this sequence: LCCA, BCA, and LSA. After the stent implantation in both the BCA and LSA, extracorporeal circulation can be discontinued and the bilateral common carotid arteries achieve hemostasis. The LSA should be fenestrated last (4, 11).

### 4 Fenestrations *in vitro*

#### 4.1 Stent graft preparation

##### 4.1.1 Direction inversion strategy

**FIGURE 3:**
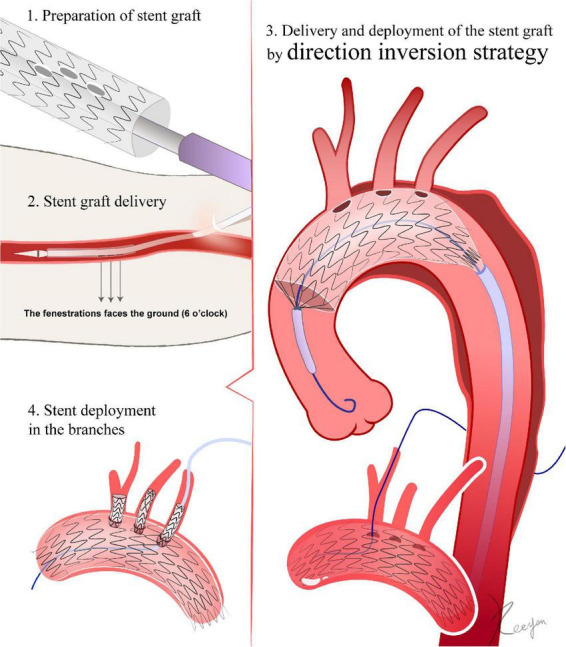
Direction inversion strategy. 1. The fenestration is made *in vitro* according to the peri-operative CTA. 2. The front of the trigger is regarded as the 12 o’clock position. When the stent graft is inserted through the femoral artery access, the 12 o’clock marker point must be turned vertically to the ground, so that it faces 6 o’clock. The stent graft is not allowed to rotate after entering the femoral artery until it reaches the aortic lesion. 3. After passing the aortic arch, the fenestration mark is aligned with the branch artery’s corresponding position. After the deployment of the stent graft, a 0.018-inch guidewire is advanced through the fenestration. 4. Deployment of stents in the branches.

Based on the preoperative CTA reconstructions, the diameter of the aorta and branch vessels, lengths, angles to the arch, clock positions, and related relationships are measured, and a preoperative design for the fenestrations is developed. The outer sheath of the stent graft is then pushed back for several centimeters under sterile conditions, allowing the proximal portion of the stent graft to be released (24). The length of the released segment should be one to two centimeters distal from the location of fenestration (24). Using a sterile ruler, the location of the fenestration is determined in accordance with the preoperative plan. The 12 o’clock position is considered to be at the front of the trigger. The position of the stent graft relative to the trigger is also referred to as the 12 o’clock position. If the fenestration must avoid stent struts, then the fenestration is deemed to be at 12 o’clock, as is the position of the trigger relative to the stent graft. The fenestration can be created using scissors or a cautery device. The fenestration can be strengthened using the loop of a snare (24, 25). To indicate the position of the fenestration during the DSA, either the original mark in the stent graft or an extra marker can be sutured to the fenestration (26) (Figure 3).

##### 4.1.2 Guidewire-assisted strategy

**FIGURE 4:**
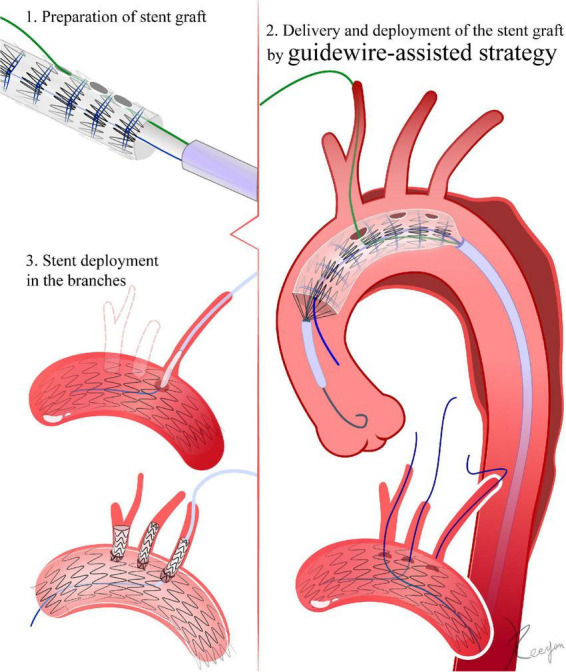
Guidewire-assisted strategy. 1. The fenestration is made *in vitro* according to the peri-operative CTA. After fenestration, a 0.018-inch guidewire passes through needle holes in the distal part of the delivery sheath into the fenestration. Diameter-reducing ties are added to the devices. 2. The 0.018-inch guidewire passes through the fenestration into the branch artery. Several segments of the stent graft are released. With the help of diameter-reducing ties, the stent graft is not fully deployed. Then, the fenestration site is aligned to the greater curve of the aortic arch by rotating the delivery system. After verifying that the fenestration is oriented toward the branch artery, the stent graft is fully released. 3. Deployment of stents in the branches.

The fenestration technique is similar to that in section “4.1.1 Direction inversion strategy (Figure 3).” After fenestration, a 0.018-inch guidewire passes through needle holes in the distal part of the delivery sheath into the fenestration. For fenestrations with more than one branch, the most proximal fenestration is preloaded with a 0.018-inch guidewire. Posterior diameter-reducing ties are added to the devices (27, 28) (Figure 4).

#### 4.2 Re-sheathed into the delivery system

The most distal portion of the stent graft is appropriately constrained by one assistant using silk thread. The outer sheath is advanced by a second assistant. The surgeon manually squeezes the stent graft back into the sheath. It should be noted that the distance between each segment should not be compressed, and the stent graft should not rotate (28–30). During the procedure, the outer sheath should not push the stent graft forward. The post-release device must not be accidentally turned on. The extra marker sewn into the stent graft should attach to the inner sheath wall (28, 31, 32). After the outer sheath has been repositioned, the stent graft needs to be flushed.

#### 4.3 Delivery and deployment of the stent graft

##### 4.3.1 Direction inversion strategy

When the stent graft is inserted through the femoral artery access, the 12 o’clock marker point needs to be turned vertically to the ground so that it faces 6 o’clock. The stent graft is not allowed to rotate after entering the femoral artery until it reaches the aortic lesion. After passing the aortic arch, the fenestration mark is aligned with the branch artery’s corresponding position. For instance, if the LCCA fenestration site is at the beginning of the first segment of the stent graft, the beginning of the first segment must be aligned with the anterior contour line of the LCCA. Once one branch artery is aligned, the remaining branches will align themselves naturally (24, 25). To prevent the stent graft from jumping forward or being pushed away, the grips need to be held and secured before the stent graft is slowly released. After the complete deployment of the stent graft, the delivery system must be retrieved and removed. Fluoroscopy can be utilized to determine whether the stent is aligned with the branch arteries. DSA should be repeated to ensure that the fenestration sites are accurate and that branch arteries are not covered (25, 26, 33).

##### 4.3.2 Guidewire-assisted strategy

Fenestrations with LSA must be used as an example. First, a guidewire passes from the LBA access to the femoral artery and exits the vascular sheath in the femoral artery. Through the guidewire, an MPA catheter is loaded from the left brachial artery into the femoral artery. A stiff guidewire (Lunderquist, Cook Medical) is positioned in the ascending aorta through common femoral access. The stent graft is advanced via the stiff guidewire, and the preloaded 0.018-inch guidewire is advanced through the MPA catheter connecting the femoral artery and LBA. When the stent graft is advanced into the descending aorta, the two guidewires that were intertwined are completely removed by rotating the delivery system. At this point, the fenestration site is aligned to the greater curve of the aortic arch by rotating the delivery system, until the O takes the shape of an I. When the stent graft reaches the aortic arch, one or two segments of the stent graft must be released. Then, the stent graft must slowly advance while constraining the guidewire connecting the LBA and femoral artery. A 6F sheath is inserted from the LBA into the fenestration via the guidewire connecting the LBA and femoral artery (27, 28). After verifying that the fenestration is oriented toward the LSA, the stent graft is fully released. In terms of fenestrations with multiple vessels, LCCA and BCA fenestrations are similar to those of the LSA. With the help of diameter-reducing ties, each fenestration is selected, and vascular sheaths are inserted into the stent graft via retrograde vascular access. The stent graft is released at the end (32, 34).

#### 4.4 Stent deployment in the branches

##### 4.4.1 Direction inversion strategy

When placing a stent into a branch artery, the guidewire can enter from the femoral artery or be introduced into the stent graft retrogradely through the branch arteries. It is necessary to make sure that the guidewire for delivering the stent is in the stent graft instead of the space between the stent graft and the aorta (24). The diameter of the stent in the branch should be the same or slightly greater (1–2 mm) than the size of the fenestration. The length of the branch stent in the aorta should be approximately 10 mm. After releasing the stent, it is necessary to verify if the stent has stenosis (24, 26). In which case, post-dilation will be needed. If the branch artery is completely covered by a stent graft, the Chimney technique may be used to restore the blood flow in the branch arteries (33).

##### 4.4.2 Guidewire-assisted strategy

During the LSA fenestration, after the stent graft deployment, the femoral artery and the LBA are connected via a guidewire. Then, a 6F-55 cm sheath is inserted into the stent graft from the LBA. After deployment, the delivery system and the stiff guidewire are removed from the ascending aorta. A stiff guidewire is used to replace the guidewire connecting the femoral artery and LBA. Then, a 12F, 80 cm-long sheath is advanced from the femoral artery into the LSA, delivering a covered stent into the LSA. The covered stent should not cover the vertebral artery orifice, and its proximal end should extend 10 mm into the stent graft. After deployment of the covered stent within the LSA, post-dilation is performed. The technique for LCCA and BCA fenestrations is comparable to that for the LSA. Through retrograde vascular access, vascular sheaths are introduced into each fenestration of the stent graft. The size of the stent placed in the branch arteries is determined by their diameter (which should be 2 mm larger than the fenestration) (34–36).

### 5 Assistive techniques and monitoring of cerebral blood supply

#### 5.1 Extracorporeal circulation

Extracorporeal circulation is recommended during all supra-aortic branch fenestrations. After the stent graft is released, all supra-aortic branches are covered. At this moment, extracorporeal circulation will provide the necessary cerebral blood supply. If the Circle of Willis is intact, the extracorporeal circulation connecting the right axillary artery and femoral vein will provide enough cerebral perfusion. Extracorporeal membrane oxygenation is also an alternative for cerebral perfusion (7, 10).

#### 5.2 Arterial bypass

There is no need for arterial bypass during LCCA and LSA fenestrations. Apart from extracorporeal circulation, arterial bypass is also an option during all supra-aortic branch fenestrations (37). A 16F sheath is inserted into the right common carotid artery and advanced to the proximal ascending aorta. An 8F short sheath is inserted into the 16F sheath. A 6F short sheath is inserted into the internal carotid artery toward the brain. The 16F sheath is connected to the vascular sheath in the left carotid artery via a vascular shunt, providing a blood supply for the left carotid artery. The proximal 8F sheath in the right common carotid artery is connected to the 6F sheath in the distal internal carotid artery, providing a blood supply for the right carotid artery (5, 6).

#### 5.3 Cerebral oximetry

Percutaneous cerebral oximetry is recommended during LCCA and BCA fenestrations, as it can monitor cerebral ischemia and hypoxia when the openings of LCCA and BCA are covered (10, 11, 25).

## Discussion and conclusion

TEVAR for thoracic aortic pathologies involving the aortic arch provides a feasible and effective approach for such diseases and has been widely used both in China and abroad, with varying results reported. However, it still faces great challenges. This Chinese expert consensus serves as a technical reference, in an effort to standardize the approach and improve the results of this procedure.

## Author contributions

CQ and ZL drafted the initial manuscript. XD, XwL, QL, XqL, WZ, PG, JP, DL, ZW, and HZ critically reviewed the manuscript. All authors approved the final manuscript.
